# Using distant supervision to augment manually annotated data for relation extraction

**DOI:** 10.1371/journal.pone.0216913

**Published:** 2019-07-30

**Authors:** Peng Su, Gang Li, Cathy Wu, K. Vijay-Shanker

**Affiliations:** 1 Department of Computer and Information Science, University of Delaware, Newark, Delaware, United States of America; 2 Center for Bioinformatics and Computational Biology, University of Delaware, Newark, Delaware, United States of America; Newcastle University, UNITED KINGDOM

## Abstract

Significant progress has been made in applying deep learning on natural language processing tasks recently. However, deep learning models typically require a large amount of annotated training data while often only small labeled datasets are available for many natural language processing tasks in biomedical literature. Building large-size datasets for deep learning is expensive since it involves considerable human effort and usually requires domain expertise in specialized fields. In this work, we consider augmenting manually annotated data with large amounts of data using distant supervision. However, data obtained by distant supervision is often noisy, we first apply some heuristics to remove some of the incorrect annotations. Then using methods inspired from transfer learning, we show that the resulting models outperform models trained on the original manually annotated sets.

## Introduction

### Background

In recent years, deep learning has achieved notable results in several fields, and there is growing interest in applying deep learning for new tasks. With the explosive growth of text in the biomedical literature, applying natural language processing (NLP) techniques and deep learning to this field has attracted considerable attention. Relation extraction (RE) plays a key role in information extraction and aids the database curation for many disciplines [1] [2] [3]. The RE task is to identify relations between entities mentioned in natural language texts and its importance in biomedical domain stems in large part due to the fact that manual curation lags behind the growth in biomedical research literature. Developing high-performing systems to automatically extract relations from text is critical, and filling an important need.

It is fairly typical to cast relation extraction as a binary classification problem: where an instance comprising of a piece of text (often a sentence) and entities mentioned in the text are annotated as positive or negative depending on whether that text expresses a relation of interest among the marked entities. While many traditional (non-deep learning) machine learning methods have been applied on these problems (see e.g. [4] [5] [6] [7]), their performance are not up to par with deep learning models when there is sufficient data.

Recently, deep learning methods have shown great improvement in the performance for various NLP tasks. Convolutional neural network and recurrent neural network are two well-studied types of deep learning architecture in NLP field. Promising results have been achieved by CNN model [8] [9] and current state-of-art CNN systems on relation extraction usually utilize refined architecture to incorporate more lexical and syntactic information. For example, in [2], piecewise max pooling process was applied after the convolutional layer to extract the structural features between the entities. The proposed method (called *piecewise CNN*) exhibits superior performance compared with pure CNN. Peng et al. [10] proposed multiple channels in CNN to incorporate the syntactic dependency information and better capture longer distance dependencies. Also, RNN model shows its advantage on relation extraction, the model in [11] achieves state-of-the-art results on protein-protein interaction (PPI) task only using the word embedding as the input of LSTM model.

However, each new task requires its own annotated data for training the deep learning model. The annotation process to develop the training data needs considerable human effort to put a label on each data instance and often requires domain expertise in specialized fields like Biomedicine. This issue is particularly onerous with deep learning since the models require setting of a large number of parameters and hence typically require large datasets. Currently, only small datasets are available for a number of tasks and this situation can hinder us from achieving the full potential of deep learning models. To address this problem, we will consider supplementing the small amount of human-labeled data with large amounts of automatically labeled data. One of the techniques to automatically label raw text data for relation extraction task is distant supervision (DS).

Distant supervision is a technique of labeling data for relation extraction utilizing a existing knowledge database. In the case of binary relation extraction, if a pair of entities are related according to an existing database then the distant supervision technique assumes that any piece of text (often a sentence) containing mentions of this pair of entities expresses the relation between them and hence labels them as positive. Mintz et al. [12] first introduced the term “distant supervision” and applied this technique to generate a large dataset for Freebase relation extraction. Before that, Craven et al. [13] had already used the relation instances (tuples) gathered from some databases to label abstracts gathered from Medline, which pioneered the distant supervision method. Since then, distant supervision has been applied on many NLP tasks. Go et al. [14] applied distant supervision to automatically classify the sentiment of Twitter messages, and Surdanu et al. [15] used distant supervision approach for the TAC-KBP slot filling task. In the biomedical field, distant supervision has also been proven to be effective on extracting protein subcellular localization [7] and microRNA-gene relations [16]. In the case of RE, distant supervision can be used to automatically obtain large training datasets using a knowledge base and large amounts of literature.

Noise in the labeling from distant supervision is a well-known problem and this labeling problem can adversely affect the performance of deep learning models [17]. To reduce the noise, many techniques have been proposed and the results show their effectiveness on the improving performance of DS-based models. One solution is to relax the originally strong assumption of DS, which assumes that all text mentioning a entity pair from the knowledge base express that relation. Riedel et al. [18] proposed the at-least-one assumption, which assumes at least one relation expression for entity pair from the DS holds, and built a multi-instance single-label model based on the DS data to reduce the noise. Then the work of [19] and [20] extended it to multi-instance multi-label model, which allows more than one label for each entity pair mention. At the same time, many other methods have also shown their advantages of reducing noise in DS data. Zheng et al. [7] introduced a threshold for the frequency of dependency paths among positive examples to filter out noisy examples. A novel generative model that directly models the heuristic labeling process of distant supervision was presented in [21]. Min et al. [22] proposed algorithm that learns from only positive and unlabeled data to alleviate the incomplete knowledge base problem. In the paper [23], the authors applied three heuristics (closest pairs, top trigger words, high-confidence patterns) to reduce the noise in the generated data, and demonstrated the improvement on performance. Since our aim is to augment the manually annotated (MA) data with large amount of DS data, we have to consider the effect of noise in DS data on the final model performance. In this work, we will use the heuristics proposed in [23] to reduce the noise in DS-generated data and explore the effectiveness of the noise reduction heuristics.

While DS data and of course, manually labeled data have been used individually to train models, we are unaware of any work that attempts use them together for training model. In this work, we will design methods to train models that use both the DS-obtained data and manually annotated (MA) data. Specifically, we consider two methods in this work: simple union and transfer learning. Transfer learning is a technique where a model (often called the source model) developed for a task is reused as the starting point for training a second (target) model on another related task [24]. The hypothesis is that since the target model starts with learned knowledge from the source model, it will achieve better performance than the models trained from a random start. Transfer learning is proven to be effective to improve the performance (see, for example [25] and [26]). It has been applied on many tasks in natural language processing with good effect [27] [28].

### Objectives of this work

To summarize, in this work, our main hypotheses are as follows. The problem of insufficient training data for deep learning models can be alleviated by supplementing manually annotated data with automatically annotated data. Give the large amount of raw text available in the biomedical domain, we are able to generate a large amount of labeled data automatically using distant supervision. However, we need to consider reducing the noise generated during distant supervision to limit its negative effect. Next, we try to design methods to combine the automatically generated data (DS data) and MA data. We hypothesize that combining these two types of data could make the model learn the knowledge from two types of data, and help the model generalize better. Specifically, we will consider transfer learning as one solution for this purpose. In addition, we also hypothesize that the learned knowledge from automatically generated data becomes more important when less manually labeled data is available.

We will test our hypotheses using two well-known relation extraction tasks in biomedical field. The first is the protein-protein interaction (PPI) extraction task [29], that is probably the most widely-studied relation extraction task in the BioNLP domain. To verify that our results generalize beyond this task, we consider second task; one of extracting protein subcellular localization task (PLOC) [30], which had previously been a focus of DS research in the BioNLP domain. For these two task, we will use two benchmark datasets as source of manually annotated data as well as the evaluation sets.

## Methods

In the section, we first introduce the overview of our method. Then the two deep learning models that are used in our experiments will be introduced. Next, we will discuss the generation of DS data and reducing the noise using heuristics in DS data. Transfer learning will also be discussed briefly. After all the basics, the details of the experiments will be illustrated. We end this section by describing some details of our experimental settings.

### Methodological overview

In this paper, we aim to augment the manually annotated data with automatically generated data by distant supervision. After acquiring the DS data besides the manually annotated data, we design two methods to combine DS and MA data: simple union and transfer learning method. In addition, the behavior of transfer learning model with less MA data is a key part of the work, which will provide us insights to build a better model when the MA dataset is small. So, the research conducted in this paper can be divided into three parts.

- Part 1: Evaluate the performance of deep learning models on DS-generated data (with and without noise reduction) and manually annotated data separately. The performance of those deep learning models will serve as the baselines of the following experiments.
- Part 2: Consider the methods of combining two types of data (DS and MA data)—simple union and transfer learning, then evaluate deep learning models performance after using combining methods.
- Part 3: This part investigates how the performance of the transfer learning process changes as the amount of MA data is changed. Specifically, we will explore how transfer learning help performance gain of deep learning models with less training MA data.

### Neural network models

In this work, we use two previously developed neural architectures that have been successfully applied on relation extraction tasks: a CNN-based model called PCNN [2] as well as a RNN-based model [11] in our experiments. Unlike the PCNN model, since the RNN model is a standard (bidirectional) LSTM model, we will call it BiLSTM model henceforth.

**Fig 1:**
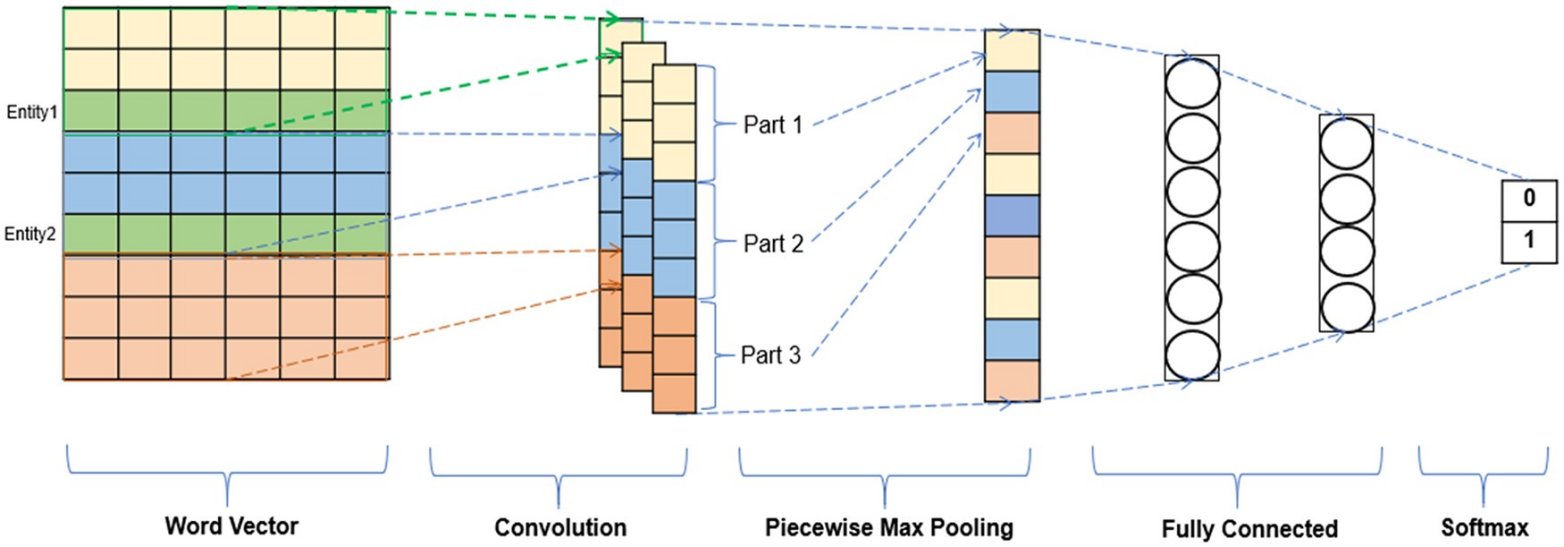
Structure of piecewise CNN model.

In this section, we will briefly introduce the architectures of the PCNN and the BiLSTM models. The CNN model for classification problem contains: 1). one convolution layer to detect the local features; 2). one pooling layer to summarize the local features; 3). one fully connected layer to classify each category; 4) a softmax layer to output a normalized probability of each category. Fig 1 shows the structure of the piecewise CNN model with the modifications to the typical situation.

In PCNN model, a sentence is divided into three parts based on the occurrences of the entities of interest in the convolutional layer. Then the max pooling in the PCNN model operates piecewise on these three parts separately. This is done in order to include more structural information about the text between the two entities. For example, consider the sentence “Among these ligands, MCP-3_PROTEIN_ had the remarkable property of binding CCR5_PROTEIN_ with high affinity without eliciting a functional response” where the entities are MCP-3 and CCR5. The three parts now are: “Among these ligands, MCP-3_PROTEIN_”, “had the remarkable property of binding CCR5_PROTEIN_”, and “with high affinity without eliciting a functional response”. The three outputs, obtained after convolution is applied on each part, are max-pooled independently. After that, these three outputs are concatenated together and form the final output of max pooling. See Fig 1, where the three parts of max pooling are shown in different colors.

**Fig 2:**
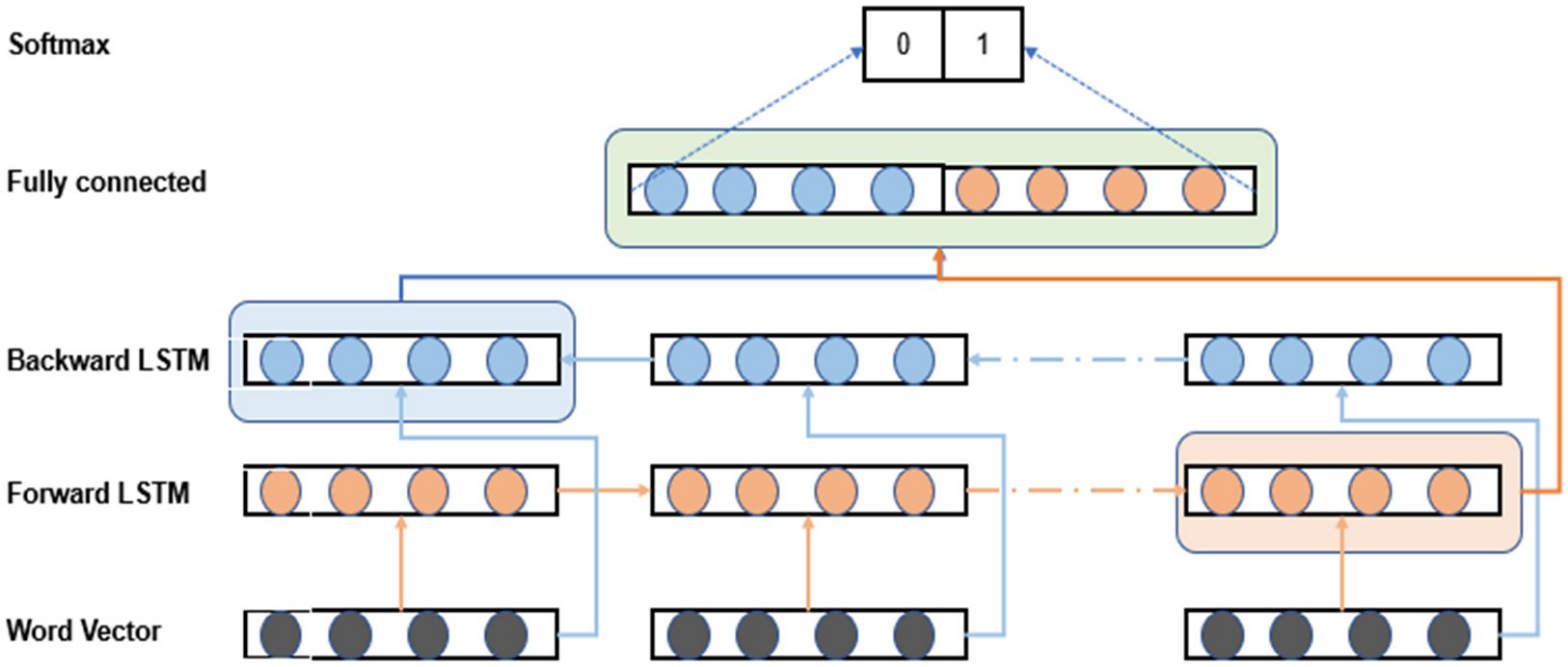
Structure of BiLSTM model.

The BiLSTM model shown in Fig 2 has: 1). an embedding layer to generate the input sequence; 2). two recurrent layers (forward and backward) to model the sequence data in bidirectional way; 3). one fully connected layer to classify each category; and 4). a softmax layer to output a normalized probability of each category.

#### Model input representation

We first represent each word by a word vector and then put all the word vectors in the same order with the sentence as our model input.

In this work, we found that both models perform better if we included more information about the input than what was included in the original papers. Specifically, in addition to the word embedding, we included the POS tag, entity type, relative distance to two entities (see below), and incoming dependency relation in the representation for each word. The original PCNN model [2] only used word embedding and positional embedding (relative distance to two entities) to represent each word, while the BiLSTM model [11] only utilized word embedding of the sentence sequence as the input vector.

In this paper, we use the word embedding pre-trained on the PubMed using skip-gram model [31]. The dimension of each word embedding vector is 200. For POS tag and incoming dependency, we extract this information from the parse results of Bllip parser [32] and covert them to unique 10-dimension vectors. The relative distances to entity 1 (*d*_1_) and to entity 2 (*d*_2_) are calculated by counting the words between the target word and the entities and the distance will be marked as negative if a word appears at the left side of the entity. After acquiring the distance numbers, we map each number to unique 5-dimension vector. From the perspective of entity type, all the words in a sentence could be divided into four types: ENTITY1, ENTITY2, ENTITY and O. ENTITY1 and ENTITY2 are the two interacting entities, ENTITY is used for the other entities in the sentence, and O stands for other words. We use a one-hot vector to represent to this feature.

### Generation of DS data

In the section, we first present the technique of distant supervision and then discuss the noise reduction methods we will adopt in our experiments. The statistics of the DS-generated datasets will be given at the end of this section.

#### Distant supervision

Recently, in the natural language processing field, distant supervision has been introduced as a method to automatically develop annotated datasets. DS assumes that the text (often a sentence) expresses a relation between entities if these entities are found in a database that captures that relationship [12]. Formally, suppose an entity pair (*e*1, *e*2) is known to be related by a relation *R* according to a database and suppose there is a sentence that mentions those two entities *e*1 and *e*2 in them. Distant supervision will label such sentences with these two entities as positive instances for relation *R* (Note that an instance is a sentence with two entities). On the other hand, if two entities are not in the database and there is a sentence containing the mentions of these two entities, the corresponding instance is taken as a negative instance for relation *R*. Utilizing this technique, we can automatically obtain large training datasets using a corresponding database for relations to label the text in literature. For example, if there is a database for the PPI relation. Assuming this database includes RFX5 and histone deacetylase 2 as an interacting protein pair. Then the following sentence will be seen as a positive instance for PPI relation between the two entities: “**RFX5** specifically interacts with **histone deacetylase 2** (HDAC2) and the mammalian transcriptional repressor (mSin3B)”. Furthermore, if the pair RFX5 and mSin3B was not included in this database as an interacting pair, then the same sentence will also be annotated as a negative sentence for the PPI relation.

The above discussion indicates that we need a database and text source for applying distant supervision. In our experiments, for the PPI task, we use the IntAct database as the interacting protein pairs database, which is a freely available, open source database system for molecular interaction data [33]. We choose the UniProt database [34] as our distantly supervised database for protein subcellular localization relation, which is a freely accessible resource of protein sequence and functional information. Medline contains abstracts for biomedical literature from around the world and it is our first choice of text source, we use it for protein subcellular localization task by randomly sampling 30,000 abstracts that contains at least one pair of protein and subcellular location within one sentence. As it is shown in [23], it gives us a skewed dataset for the PPI task– positive/negative ratio is 1: 7.4. In order to acquire more balanced positive and negative instances for PPI, we just use the literature found in the IntAct database as our text source (Positive:Negative = 1:1.5).

#### Noise reduction heuristics

Distant supervision labeling process is noisy. There can be noise in the positively labeled data since the DS process will label a sentence as positive if the sentence mentions two entities that are stored in the database for this relation. Thus, the actual content of the sentence is not taken into account while labeling the sentence as positive. For example, the sentence “These data suggest that the functions of the Cul2-**Rbx1** and **Cul5**-Rbx2 modules are distinct.” will be wrongly labeled as positive by DS even though there is no relation between these two highlighted entities. Also, distant supervision can also introduce mistakes while labeling a sentence as negative. Errors of this kind are due to the incompleteness of the relation database being used. For instance, DS will label the sentence “RFX5 specifically interacts with histone deacetylase 2 (HDAC2) and the mammalian transcriptional repressor (mSin3B), whereas **RFX1** preferably interacts with HDAC1 and **mSin3A**.” as negative when the database does not contain a PPI relation between RFX1 and mSin3A, however the sentence seems to suggest otherwise.

We considered a number of heuristics that have been proposed for DS noise reduction. We eventually decided to use the ones chosen in the work of [23], as it obtained good results. These heuristics, explained below, are the Closest Pairs (CP) and the Trigger Words (TW) heuristics applied on the positive instances and the High-confidence Patterns (HP) heuristic applied on negative instances.

**Closest Pairs:** Consider the sentence “The interaction between **bICP0** and **IRF7** correlates with reduced trans-activation of the IFN-beta promoter by IRF7”. Further suppose a PPI database contains the PPI relation between bICP0 ad IRF7. Thus, this sentence will be annotated twice as a positive instance, once with the bOCP0 and the first instance of IRF7 and the second time with bICP0 and the second occurrence of IRF7. The first instance does indeed appear to be correctly annotated by this process, however an inspection of the second instance will cast doubt about the second instance as specifying the PPI relation between the two entities involved. This kind of situation where an entity is mentioned multiple times in a sentence is not uncommon in biomedical literature and invariably only on the occurrences may be involved in a specific relation. The closest pair heuristic is applicable when there is a sentence that contains a pair of entities where at least one of them is mentioned multiple times in the sentence. Only one of the instances for this sentence will be consider positive, the instance where the pair of mentions of the entities are closest to each other in the text. The others are not labeled negatively but rather these instances are just removed from the dataset.

**Trigger Words:** Typically a ‘relation’ trigger word is used to express the relation in a sentence. These kind of trigger words are usually verbs, but sometimes in their nominal or adjectival form. For example, ‘bind’ is the trigger word in the sentence “Two-hybrid data and other genetic evidence suggest that **Rpa43** directly bind **Spt5**”. Using a heuristic to automatically guess if a sentence contains a trigger word for the relation. Then we remove all the DS-generated positive instances if they do not contain trigger word. Details of automatically identifying a trigger word for a relation can be found in [23].

**High-confidence Pattern:** Most knowledge database are likely to be incomplete and not record all the interacting entity pairs. Sentences that indicate a relation between entities will still be labeled as negative if the pair of entities is not contained in the database. The high-confidence pattern heuristic was introduced to handle this kind of noise from the negative instances. After the first round of distant supervision, if some patterns appear repeatedly in many positive instances, we could consider them as high-confidence patterns of expressing a relation. Those high-confidence patterns on the positive instances can be used to remove the wrong-labeled negative instances. For example, the sentence “This indicates that **AtGRIP** and **AtARL1** interact directly.” is labeled as negative by DS, but it is obviously positive. After applying the high-confidence pattern, it will be removed from the negative instances because it follows the frequently occurring pattern “PROTEIN 1 and PROTEIN 2 interact” among DS-labeled positive instances.

We find that the definition of trigger word in the original paper is the ‘verb’ (or in nominal/adjective form) that expresses the relation between two entities, but the related two entities do not have verbal trigger word in many cases in PLOC task. So we only apply heuristic CP on positive instances for the PLOC task. Since we only apply two heuristics on PLOC DS dataset, we further filter out the noise by choosing the top 20 location names based on their frequency.

#### DS corpora statistics

In this section, we will discuss the creation of different DS datasets for each task. We have already stated the required knowledge base and text source for distant supervision, the last thing we have to consider is to detect all the entity names (protein and subcellular location names) in the biomedical literature.

For detection of protein names, we utilize the output of GNormPlus [35], which is an end-to-end system that detect gene/protein names. For the subcellular location names, we use location names from UniProt as a dictionary to match the mentions in the Medline text.

The first row in Table 1 shows the number of positive and negative instances we used for DS data for the two tasks. The next 5 rows show the size after applying different heuristics and their combinations.

**Table 1:** DS data and test data statistics.

PPI			PLOC		
Dataset	Positive#	Negative#	Dataset	Positive#	Negative#
RAW	54,170	82,517	RAW	19,654	32,254
CP	38,644	82,517	CP	15,519	32,254
HP	54,170	77,559	HP	19,654	30,206
CP+TW	25,294	82,517	CP+TW	-	-
CP+HP	38,644	77,559	CP+HP	15,519	30,206
CP+TW+HP	25,294	77,559	CP+TW+HP	-	-
AIMed	1,000	4,834	LocText	351	338

Baseline: original DS-labeled data without any heuristic; CP: Apply closest pair heuristic on DS data; HP: Apply high-confidence pattern heuristic on DS data; CP+TW: Apply closest pair and trigger word heuristics on DS data; CP+HP: Apply closest pair and high-confidence pattern heuristics on DS data; CP+TW+HP: Apply closest pair, trigger word and high-confidence pattern heuristics on DS data.

### Transfer learning

Recall that we will employ transfer learning to combine the DS-labeled data and manually annotated data. In this section, we will explain why transfer learning is a feasible approach to learn knowledge from two different datasets for same task.

Transfer learning focuses on storing knowledge gained while solving one problem and using it to a different but related problem. It has been successfully applied in many cases [24]. One standard way of using transfer learning is to train a model for one task and then this pretrained model can be used as a starting point for training on a dataset for a related task. In this paper, we consider the model trained on DS data as a source model and transfer knowledge gained to the task which is defined by the MA data. Thus, we consider the underlying tasks defined by DS data and MA data are two similar but different, related tasks. The DS data for PPI and PLOC relation extraction task is generated without paying any attention to the text. On the other hand, the MA data is labeled by human based on their semantic understanding of the relation and the sentence. So we can see that the models trained on these two datasets are learning (perhaps) slightly different knowledge. Thus, we can take the two models built on DS and MA data as two different models for closely related tasks.

### Experiments conducted

In this section, we will conduct experiments to address different problems in the three parts of research work (from methodological overview) regarding the use of DS-data to augment manually annotated data. The first set of experiments involve developing deep learning model on DS and MA data separately, and giving us baselines whose performance provides context to compare and interpret the results of the next two sets of experiments. The effect of noise-reduction on DS data is also investigated. The second set of experiments focus on the main concern of this work: design methods to combine DS and MA data.

Our motivation in this work is to supplement manually labeled data especially when it might not be sufficient for effectively training deep neural models. In our final set of experiments, we try to experimentally determine how much DS-derived data can compensate for small size of manually labeled data.

#### Experiment 1: Baseline models and DS noise reduction

After acquiring a large amount of DS data, we start off by investigating the performance of models trained on only the automatically derived DS data. Different from [23] which uses a logistic regression model, we investigate the performance of our deep learning models with and without the application of noise-reduction heuristics on the DS data.

The human-labeled data for the two tasks can also be used to train the models. These models will be evaluated on these labeled sets using 10-fold cross-validation. The models discussed here, i.e., the ones trained on DSO (original DS data), DSNR (DS data with noise reduction) and MA can serve as our baseline models to evaluate the models discussed in the following subsections.

#### Experiment 2: Data combining methods

As we have large DS-labeled datasets available, an obvious question is to consider how to combine it with the manually annotated dataset. The most straightforward way to combine two datasets is to simply take the union of DS data and MA data, and we will use it as a baseline here. We will also employ transfer learning as discussed previously to combine DS-obtained and MA data in this paper. Specifically, we pre-train the model on (noise-reduced) DS datasets, then fine tune the model on MA training set to further adjust the parameters of the model.

In the pre-trained model, the learned knowledge of data stores in the hidden layers’ weights. These weights mean convolution filter (feature map) weights and the fully connected layers weights for CNN model, meanwhile mean recurrent cell weights and the fully connected layer weights in RNN model. Since the fully connected layer weights play the role of classifying the label of instance based acquired features in theory, convolution filter weights and recurrent cell weights contain the most important information learned from pre-training data. In this paper, we do not eliminate fully connected layers weights directly, since their functionality is not well studied. Instead, we design two options for transfer learning: 1). only transfer the convolution filter weights/recurrent cell weights; 2). transfer both convolution filter weights/recurrent cell weights and fully connected layer weights.

**Fig 3:**
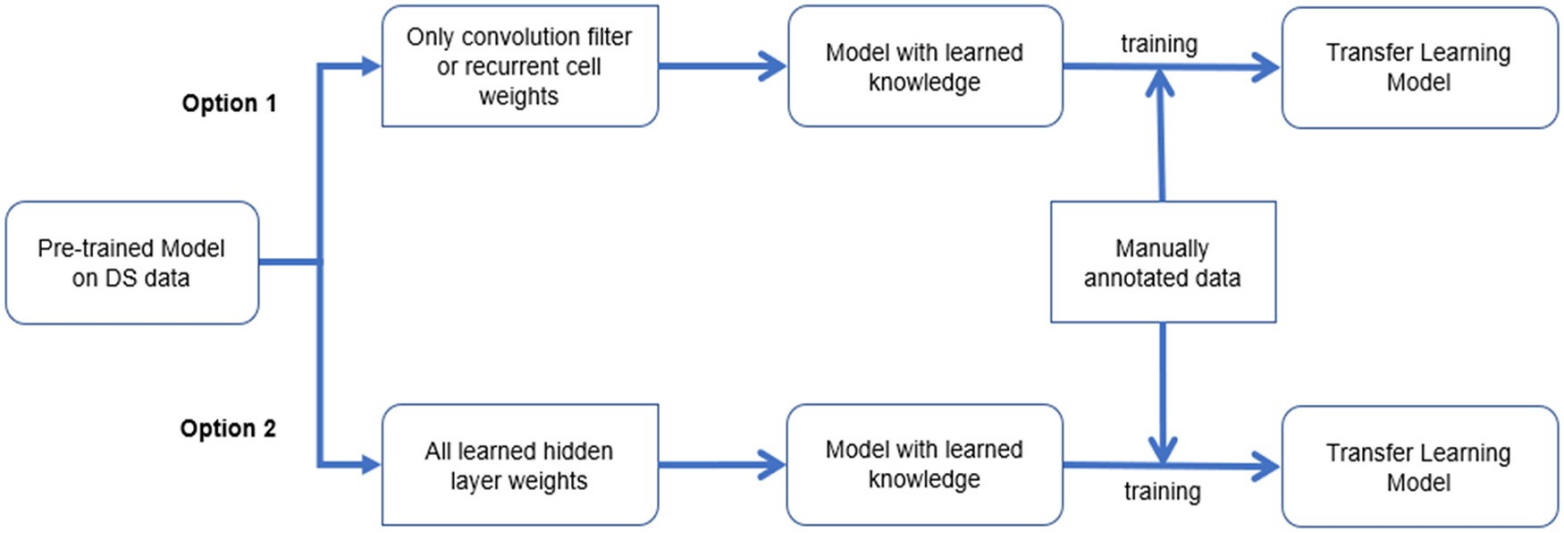
Pipeline of transfer learning model on both DS data and human-labeled data.

Fig 3 shows the pipeline of transfer learning model. When we use manually annotated data in both training and test process, we will perform cross validation to obtain the final results.

#### Experiment 3: Impact of size of MA training data

The motivation for distant supervision is to improve performance when there is only a limited amount of human-labeled data. Thus, it is worth examining the impact of the size of manually annotated data on the performance. For this set of experiments, we obtain transfer learning models pretrained on DS data and then use different sizes of manually annotated data to evaluate the dataset size effect. Specially, we will utilize 25%, 50% and 75% of the manually annotated data in the transfer learning training process to evaluate the performance of models.

### Experimental setup choices

#### Parameter choices

We implement the models with Tensorflow [36], and the maximum length of sentence is set to 100, which means the longer sentences are pruned and the shorter sentences are padded with zeros. The batch size is 128 during model training. The convolution filter number is 400 and the learning rate is 0.001 for PCNN model. Also, we apply decayed learning rate on PCNN with 0.95 decay rate and 1000 decay steps. For BiLSTM, we set the hidden state dimension to be 400 and utilize constant learning rate of 0.001. We also apply dropout in these two models with drop rate of 0.5 on convolution/recurrent layer(s), and drop rate of 0.2 on dense layers. The training epoch is 30 for the DS and mixed data (DS+MA) for both models, which is the compromise of between the performance on mixed data and training time (more training epochs achieve slightly better results but need longer training time). The training epoch on MA data and transfer learning MA data is 200 for PCNN and 100 for BiLSTM (BiLSTM is trained with less epochs since it needs more time to train). Plus, the window size for PCNN is 3.

#### Evaluation sets

AIMed [37] is a widely used benchmark dataset for PPI task, we will use it as our evaluation set for PPI. LocText corpus [38] will be our evaluation set for PLOC task, which is a well annotated dataset with tagtog tool [39]. Please see the last row of Table 1 for the statistics of these two corpora.

## Results and discussion

Throughout this section, we use precision, recall, F1 score as measurement to evaluate the performance of deep learning models.

### Models trained on DSO, DSNR and MA corpora

Although we differ from [23] in the choice of models which used Logistic Regression and Naive Bayes, we observe the same type of patterns when using the noise-reduction heuristics to filter the raw DS set (DSO). Other than some minor differences (e.g. precision of CP+TW+HP), the performance of the deep learning models are noticeably higher here.

The model built on noise-reduced DS data should achieve better performance as the heuristics on positive and negative instances will improve the precision and recall respectively. Heuristics that reduce the noise in positively-labeled instances have the potential to improve the precision of the resulting models. Likewise, addressing noise problem in negative instances should help improve recall.

**Fig 4:**
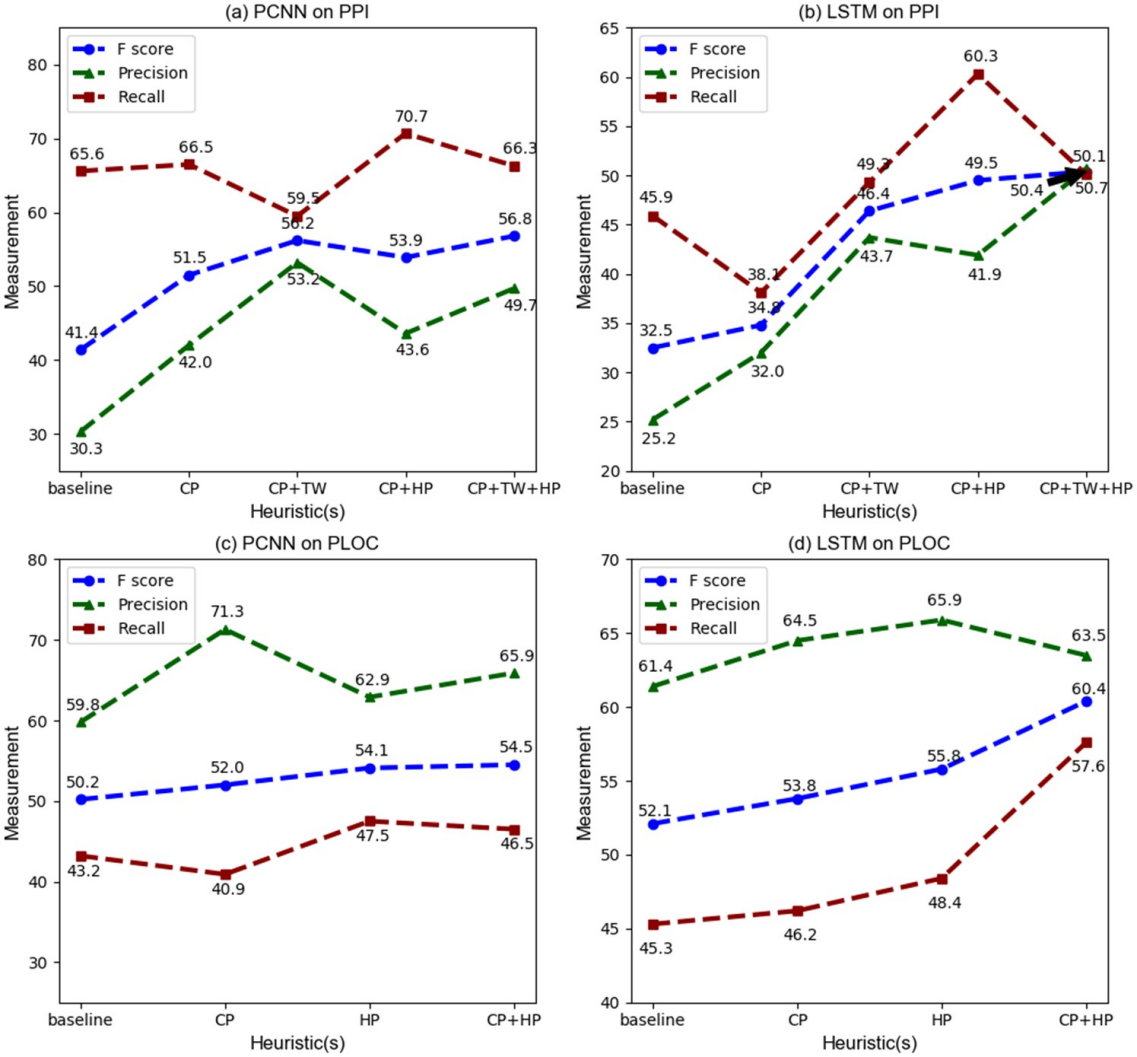
Performance of models built on DS data.

Fig 4 shows the results of learning of the two types of neural network architectures on the two tasks. As is to be expected, precision improves with the use of CP, with the increase more noticeable in the PPI task case. Despite the drop in recall in BiLSTM-PPI case, the F1 score improves in all four cases.

Next, the application of TW is considered and the expected increase in precision is noticed in both PPI graphs. As we discussed before, it is not proper to apply TW heuristic on PLOC dataset. In the PLOC case, we only see the improvement of precision on the use of CP heuristic.

Finally, the addition of HP boosts the recall in all four cases. Thus, we see that the addition of these noise-reduction heuristics helps boost the performance with F1 score showing an increase of 4% to 18%. In fact, in the case of PCNN on the PPI data, the performance on AIMed with supervised learning on DS data is comparable to leading results obtained previously prior to the use of neural network models [10]. While the PCNN model obtains better results on the PPI task, the BiLSTM-based model performs better on the PLOC task, when trained on DS (with noise reduction) data.

Finally, we report the results for the same four combinations but this time using manually annotated data for training. As noted earlier, these results are based on 10-fold cross validation on the manually annotated sets for the two tasks. Row *Model*_*MA*_ of Tables 2 and 3 shows the performance of the regular supervised learned models. Notice that the PCNN model achieves better F1 scores due to better recall results for both tasks, although the BiLSTM model has higher precision. Supervised learning on MA data improves the F1 score between 13% to 24% over noise-reduced DS trained model.

**Table 2:** Results of deep learning models on PPI.

PCNN				BiLSTM			
Model	Precision	Recall	F score	Model	Precision	Recall	F score
*Model*_*DSNR*_	49.7	66.3	56.8	*Model*_*DSNR*_	50.7	50.1	50.4
*Model*_*MA*_	75.6	76.1	75.8	*Model*_*MA*_	78.1	69.7	73.7
*Model*_*Mix*_	65.0	68.2	66.5	*Model*_*Mix*_	64.2	54.9	59.2
*Model*_*TL*_*CON*_	76.7	79.3	78.0	*Model*_*TL*_*REC*_	78.3	74.5	76.4
*Model*_*TL*_*ALL*_	77.1	81.2	**79.1**	*Model*_*TL*_*ALL*_	78.9	74.7	**76.8**

*Model*_*DSNR*_: model built on noise-reduced DS data; *Model*_*MA*_: model built on manually annotated data (AIMed for PPI and LocText for PLOC); *Model*_*TL*_*CON*_: transfer learning using only the convolutional features; *Model*_*TL*_*REC*_: transfer learning using only the recurrent cell features; *Model*_*TL*_*ALL*_: transfer learning using all the pretrained parameters.

**Table 3:** Results of deep learning models on PLOC.

PCNN				BiLSTM			
Model	Precision	Recall	F score	Model	Precision	Recall	F score
*Model*_*DSNR*_	65.9	46.5	54.5	*Model*_*DSNR*_	63.5	57.6	60.4
*Model*_*MA*_	74.1	83.8	78.6	*Model*_*MA*_	76.0	70.5	73.2
*Model*_*Mix*_	71.4	69.9	70.6	*Model*_*Mix*_	72.7	63.7	67.9
*Model*_*TL*_*CON*_	75.3	82.1	78.6	*Model*_*TL*_*REC*_	79.2	78.3	78.8
*Model*_*TL*_*ALL*_	76.1	84.9	**80.3**	*Model*_*TL*_*ALL*_	80.3	78.4	**79.4**

### Combining DS and MA data

This subsection is concerned with the core question of this work: how much improvement can we obtain by augmenting manually annotated data with (noise reduced) DS data. Tables 2 and 3 show the results of various models. The first two rows are only repetitions of the results from previous subsection and provide the context for the new results using combined training data. The first row corresponds to the model *Model*_*DSNR*_ obtained by training on noise-reduced DS data, whereas the second row corresponds to the model *Model*_*MA*_ obtained by training on purely manually annotated data. As mentioned earlier, the pure data combination is the simplest way to utilize these two datasets, and hence we first consider the union of human-labeled data and (noise reduced) DS data. The third row of Tables 2 and 3 shows the performance of the resulting trained models, designated *Model*_*Mix*_. The drop in the performance observed by comparing the second row suggests that simply taking the union of the instances on the two data sets may not be an appropriate way of augmenting the manually annotated data. Both precision and recall drop in all four cases. We hypothesize that the drop in performance might be due to some remaining noise in the DS data and/or that there might be some additional constraints in the manual annotation guidelines that might not be captured in the DS data.

In the next two rows of Tables 2 and 3, we display the performance for the two options that we have used for transfer learning: 1). *TL*_*CON*/*REC*_: transfer learning with only convolution filter weights or recurrent cell weights from pre-trained model; and 2). *TL*_*ALL*_: transfer learning with all the weights of pre-trained model.

These two tables shows that both transfer learning models perform better than the models built on DS-labeled data as well as human-labeled dataset (AIMed and LocText). In fact, as hoped, the performance exceeds that of all other models, and obtains the best results ever. This implies that the deep learning models learn the knowledge in both DS and human-labeled data, and even though there may still be noise in DS data, the transfer learning process utilizes the human-labeled data to remedy the mistakes before and lead the learning in right direction in the second phase of model training. Thus, transfer learning is an effective way to make the best of DS labeled data and limited human-annotated data.

For the two options of transfer learning, we notice that the way of transferring all weights of pre-trained model obtains slightly better results overall. Thus, transferring all weights is our default way of transfer learning in our following experiments.

### Effect of human-labeled dataset size

Any potential gains of the data augmentation method are more meaningful when the amount of available human-labeled dataset is not large. However, this is also a situation where any noise in DS derived data discrepancy between it and human-labeled data might hamper the effectiveness of data augmentation with DS data. This motivated the third set of experiments where we use noise-reduced DS data (and transfer learning) in conjunction with 25%, 50%, 75% of the human-labeled data in the model training process.

**Fig 5:**
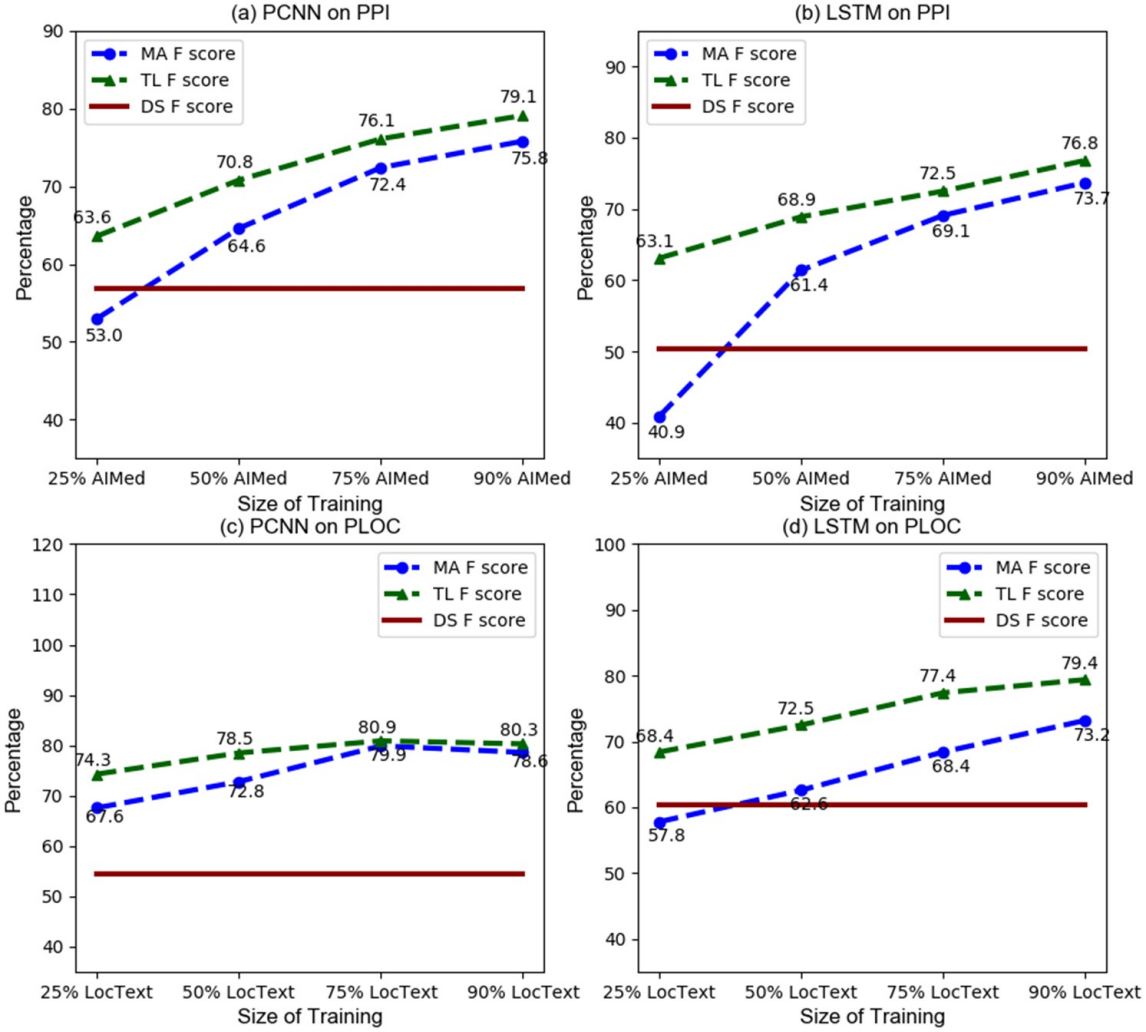
Trend of F score with different size MA data in transfer learning. MA F score means the F score acquired from models built on MA data only; TL F score means the F score acquired from models built on transfer learning; DS F score means the F score acquired from models built on DS data.

Fig 5 shows the F1 score corresponding to different sizes of human-annotated data, where 90% case corresponds to the results from previous subsection. The performance of the model obtained using transfer learning is shown and compared with those obtained with just the human-annotated (of the same size) data and with DS data. For example, training on 25% of AIMed data on the PPI task, the transfer learning method enables us to improve the performance by 10.6% and 22.2% using PCNN and BiLSTM respectively over the models of training on corresponding size of human-labeled data alone. We believe this shows reasonably good performance can be achieved with just 25% of manually labeled data using transfer learning, especially compared to using manually labeled data alone. Notice that with 25% of the data, the performance of the model trained on manually labeled data is worse than the model trained using DS data alone in three cases out of four. The improvement using transfer learning narrows as the size of the human-labeled data increases. Improvement is also seen on the PLOC data, although the improvement is less than what was obtained for the PPI task. These results show that transfer learning and data augmentation approach always improves over the training on manual data alone, with the larger improvement shown when the size of human-labeled data is smaller, i.e., when there is limited human-labeled data, a situation which motivates this work.

**Fig 6:**
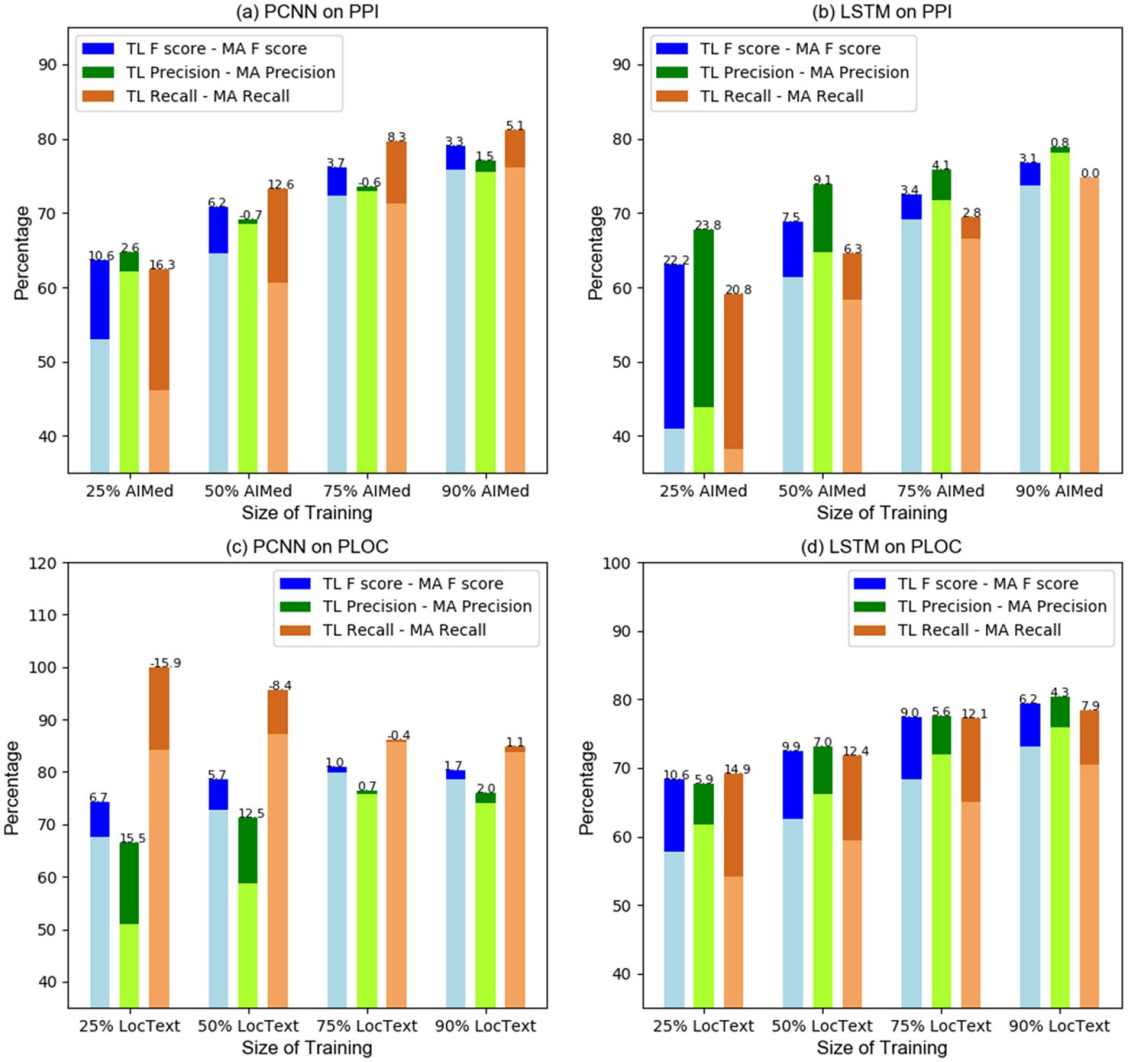
Size effect of human-labeled dataset. The number on each bar stands for the difference between None Transfer Learning and Transfer Learning model. Positive number means Transfer Learning improves the metric, while negative number means Transfer Learning deteriorates the metric.

Fig 6 additionally presents the precision and recall numbers for more detailed analysis. For the PPI task with smaller amount of human-labeled data, most of the gains of transfer learning over just human-labeled data training are due to improvement in recall, although for BiLSTM-based model, the gains in precision are also substantially resulting in higher F1 score gain. With PLOC case, the gains in precision and recall are noticed.

## Conclusion

In order to improve the performance of deep learning models on small datasets, we have considered augmenting them with automatically obtained datasets using distant supervision. We show that some heuristics can be used to alleviate the well-known noisy annotation issue with distant supervision. Improvement of performance of both PCNN and BiLSTM models on both tasks is obtained.

Two methods of utilizing both DS data and manual data are discussed. Mixing DS data and human-labeled data to obtain the training data for deep learning model is the simplest way to combine data, but the performance does not show improvement over using human-labeled data alone. However, we show that the mechanism of transfer learning provides much better results than either of these two types of data individually.

We also explore the feasibility of reducing the size of manual data with the availability of large DS dataset. It can be seen that impact of transfer learning is much more beneficial when the manual data size is small (F score increased 10.6% when using 25% of AIMed). So when developing large human-labeled dataset is not feasible, applying transfer learning on DS data becomes more important.

These results are obtained for both types of deep learning models as well as both tasks, we plan to apply this technique on other relation extraction tasks. We will continue to pursue other heuristics to further reduce the noise in the automatic corpus creation with DS. Given the imbalance in the distribution of positive/negative instances in these datasets, we plan to conduct additional research to address this issue.
